# Antibiotic surveillance on a paediatric intensive care unit: easy attainable strategy at low costs and resources

**DOI:** 10.1186/1471-2431-12-196

**Published:** 2012-12-21

**Authors:** Martin Stocker, Eduardo Ferrao, Winston Banya, Jamie Cheong, Duncan Macrae, Anke Furck

**Affiliations:** 1 Paediatric and Neonatal Intensive Care Unit, Children’s Hospital Lucerne, Lucerne, Switzerland; 2 Paediatric Intensive Care Unit, Royal Brompton and Harefield NHS Foundation Trust, London, UK; 3 Epidemiology and Biostatistics, Royal Brompton and Harefield NHS Foundation Trust, London, UK; 4 Pharmacy Department, Royal Brompton and Harefield NHS Foundation Trust, London, UK

**Keywords:** Antibiotic surveillance, Paediatric intensive care unit, CDC 12-Step Campaign, Checklist, antimicrobial stewardship program

## Abstract

**Background:**

Antibiotic surveillance is mandatory to optimise antibiotic therapy. Our objectives were to evaluate antibiotic use in our pediatric intensive care unit (PICU) and to implement a simple achievable intervention aimed at improving antibiotic therapy.

**Method:**

Prospective, 3 months surveillance of antibiotic use on PICU (phase I) and evaluation according to the CDC 12-step campaign with development of an attainable intervention. 3 months surveillance (phase II) after implementation of intervention with comparison of antibiotic use.

**Results:**

Appropriate antibiotic use for culture-negative infection-like symptoms and targeted therapy for proven infections were the main areas for potential improvement. The intervention was a mandatory checklist requiring indication and recording likelihood of infection at start of antibiotic therapy and a review of the continuing need for therapy at 48 h and 5 days, reasons for continuation and possible target pathogen. The percentage of appropriate empiric antibiotic therapy courses for culture-negative infection-like symptoms increased from 18% (10/53) to 74% (42/57; p<0.0001), duration of therapy <3 days increased from 18% (10/53) to 35% (20/57; p=0.05) and correct targeting of pathogen increased from 58% (7/12) to 83% (20/24; p=0.21).

**Conclusions:**

Antibiotic surveillance using the CDC 12-step campaign can help to evaluate institutional antibiotic therapy. Development of an attainable intervention using a checklist can show improved antibiotic use with minimal expense.

## Background

Antibiotic surveillance is mandatory to optimise antibiotic therapy and to prevent antimicrobial resistance [1-3]. Guidelines for developing an institutional program to enhance antimicrobial stewardship are published and contain several interventions with good impact but require a high level of organisational change, resources and acceptance [4]. Only a small number of studies have focused on antibiotic surveillance on hospitalised newborns, infants and children [5-8]. In a recent published survey, Hersh et al. pointed out that lack of resources, including funding, time, and personnel were the major barriers to implementing an antimicrobial stewardship program in paediatrics [9].

The Center for Disease Control and Prevention (CDC) 12-step program to prevent antimicrobial resistance was launched in 2000 to educate clinicians about antimicrobial resistance and provide strategies to improve clinical practice [10]. Patel and colleagues showed in a recent publication the possibility of using the CDC 12-Step Campaign to assess antibiotic prescribing and adherence to guidelines on a Neonatal Intensive Care Unit (NICU) [8].

In a recently published concise review, Newland and Hersh concluded that prospective audit with feedback was the most favourable and efficient strategy for antimicrobial stewardship programs in paediatrics [11]. They asked for more publications on prospective audits and declared the most important challenge was to develop strategies to facilitate greater implementation of antimicrobial stewardship programs at low cost and low resource requirement. In line with these conditions our aim was to conduct an antibiotic surveillance audit on our Paediatric Intensive Care Unit (PICU) with a simple achievable strategy at low cost and low resource requirement.

## Method

We performed a prospective, single-centre interventional audit of antibiotic use on our PICU. Antibiotic therapy was assessed looking for adherence best practice recommendations of the CDC 12-step campaign. Basic data collection was carried out for 3 months (phase I). Interventions were then planned based on areas for potential improvement, that were simple and attainable, required minimal personnel resources and were low in cost (no funding available). Data collection was repeated after the intervention based on the power calculation (phase II). The audit was supervised by the departments of Paediatrics, Pharmacy and Microbiology at the Royal Brompton and Harefield NHS Foundation Trust. The results of phases I and II were reviewed as part of the clinical governance program. This audit with an implemented intervention that is part of standard care on our PICU without a change in policy didn't required ethical approval.

### Site and subjects

This audit was conducted on the PICU at the Royal Brompton and Harefield NHS Foundation Trust in London, UK. Our unit is a specialised, tertiary unit for paediatric cardio-respiratory care with approximately 700 admissions per year. Newborns and children up to 16 years of age with a broad spectrum of cardiac malformations, cardiac diseases, rhythm disturbances, postoperative cardiorespiratory care and respiratory problems are treated in our facility. All admissions between 1^st^ April 2010 and 31^st^ June 2010 (91 days) were included in phase I, and then between 10^th^ November 2010 and 28^th^ February 2011 (110 days) in phase II.

Staff of the unit includes physicians (intensivists, cardiologists, respiratory physicians, anaesthesists, cardiothoracic and general paediatric surgeons) working as consultants, fellows and senior house officers; nursing staff working usually in 1:1 care (1 nurse / 1 patient), partially 1:2 if patients were stable and not invasively ventilated; pharmacists and allied health professionals. The group of physicians prescribing antibiotics remained unchanged in regard of number and level of expertise during both study periods: 5 consultant intensivists, 9 fellow intensivists and 9 senior house officers (Paediatrics). Infection control is part of our standard practises on our unit and two infection-focused care bundles are actively practised with prospective data collection: i) care bundle for prophylaxis of catheter-related infections, ii) care bundle for prophylaxis of ventilator-associated pneumonia. Both care bundles remained unchanged and there were no other stewardship interventions in place during the study phase.

### Data collection

Data collection in both phases was carried out using the electronic prescribing system for medication (ICIP), the electronic microbiology data system (EPR), the electronic system for nursing reports and all available medical reports. With the electronic medication and microbiology data system we were able to assess all doses of antibiotic therapy administered to patients on PICU, and results of all microbiologically tested samples (blood, cerebrospinal fluid, ascites, urine, tracheal aspirates). Indications for antibiotic therapy were obtained from medical and nursing reports in phase I, and additionally from the study intervention form during phase II.

### Definitions and assessment of antibiotic use

The design of our study was an antibiotic surveillance audit, based on the CDC 12-step recommendations focused at the 2 strategies “Diagnose and treat infection effectively” (steps 3–5) and “Use antimicrobials wisely” (steps 6–10). The unit of antibiotic analysis was an antibiotic course, defined as one or more antibiotics given concurrently for one or more days. The possible reasons for therapy were categorised as 1) prophylaxis (post-surgery), 2) empiric therapy (clinical signs of possible infection with negative cultures) or 3) therapy for proven infection (clinical signs of infection with positive cultures from blood, cerebrospinal fluid, ascites, urine or tracheal aspirates). Appropriateness of antibiotic use was compared using institutional antimicrobial guidelines for prophylaxis and empiric therapy for suspected infections. Appropriate duration of empiric therapy for culture negative infection-like symptoms was defined as less than 3 days unless there were clear and reasonable documented reasons for continuing antibiotic therapy.

### A checklist as a simple achievable intervention

**Figure 1:**
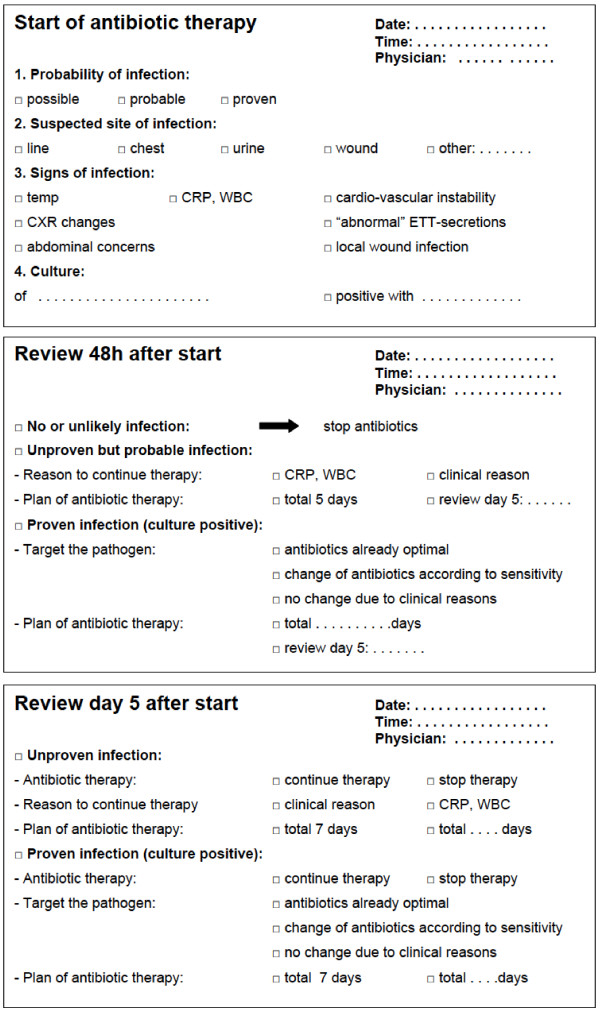
Checklist for antibiotic stewardship.

After defining 2 main areas for potential improvement in phase I, we created a checklist as a simple achievable intervention (Figure 1). The checklist was introduced to the multidisciplinary team of the PICU without further education regarding antibiotic prescribing policy. The intervention was a mandatory form (one page per antibiotic course) for all children receiving antibiotics (except prophylaxis). The checklist requested indication and the likelihood of infection at the start of antibiotic therapy, review of therapy at 48 hours and 5 days, recording reasons for continuing therapy and targeting therapy towards the pathogen if cultures were positive. The likelihood of infection estimated by the treating physician was scaled. This initial impression was intended to guide subsequent in deciding whether antibiotic therapy could be stopped after 48 hours. The forms were reviewed and updated twice daily by the treating physician (fellow or consultant intensivist). There was no strict monitor system regarding compliance and adherence to the intervention. Pharmacists working on the ward controlled at random use of the checklist and approached physicians not following the instructions.

### Statistics

A power calculation was done based on the aim of our primary intervention, which was to reduce inappropriate duration of empiric antibiotic therapy with culture negative results. In phase I, 18% (10/53) of patients on empiric therapy received it for an appropriate duration. We hypothesised, that it was possible to increase this to 36% (duplication) with the implemented intervention. With the definition of alpha=0.05 and a power of 80%, 42 courses of empiric therapy would be required to demonstrate a significant result. Calculating a possible compliance of 80% to the intervention our aim was to observe 52 courses of empiric antibiotic therapy during phase II. A Chi-squared test was calculated if all frequencies were equal to or greater than 5, otherwise a Fisher’s Exact two-tailed test was used.

## Results

### Results of phase I (baseline period)

Assessing antibiotic use according to the CDC 12-step campaign revealed 2 main areas in phase I for potential improvement: i) Step 10: Empiric antibiotic therapy for < 3 days for unproven infections (culture negative); ii) Step 4: targeting of pathogens as therapy for proven infections (culture positive). 18% (10/53) of empiric antibiotic courses had a duration of < 3 days and there was no clear and reasonable documentation for courses of longer durations. In 58% (7/12) a cultured pathogen was targeted with a change of antibiotics unless antibiotics were already susceptible. The other CDC steps showed good results, i.e. 95% of all prophylactic courses complied with institutional guidelines and vancomycin, carbapenems and third generation cephalosporins were rarely used and did not appear in the top 5 most commonly used antibiotics (results not shown).

### Results of phase II (intervention period)

Baseline comparisons of patient demographics, overview of antibiotic courses and outcomes are shown in Table 1. There were significantly more proven infections during phase II (24/182 against 12/194 during phase I; p=0.02), but empiric antibiotic courses with negative culture results were similar (Tab 1). After implementation of the checklist in phase II, there was an increase in the empiric antibiotic courses < 3 days from 18% (10/53) during phase I to 35% (20/57; p=0.05). In additional a further 40% (22/57) had clear and reasonable documentation about reasons for continuing antibiotic therapy despite negative culture results (clinical signs and/or significantly raised infection markers). Therefore in phase II, 74% (42/57) of empiric antibiotic courses were classified as appropriate (p<0.0001). The percentage of correctly targeted pathogens increased from 58% (7/12) in phase I, to 83% (20/24) in phase II (p=0.21). The checklist was used during phase II in 56 out of 81 courses of empiric or proven antibiotic therapies (compliance rate of 69%).

**Table 1:** Baseline demographics, overview of antibiotic courses, outcome variables

	**Phase I (91 days)**	**Phase II (110 days)**	**p-value**
Admissions	174	185	0.48
Hospitalisation days	952	1186	0.84
Hospitalisation days / admission^a^	5.47 (1/3/41)	6.41 (1/3/71)	0.24
Fatal casualities	0	3	0.25
Infection-related fatal casualities	0	0	1
Sex (% male)	58%	60.5%	0.23
Age at admission^b^	6 mt (0–16 yrs)	7 mt (0–16 yrs)	0.48
Cardiosurgery admissions	67.8%	57.8%	0.05
Cardiology admissions	13.2%	20.5%	0.06
Respiratory admissions	10.3%	13.5%	0.35
Other admissions^c^	8.6%	8.1%	0.86
Antibiotic courses total	194	182	0.14
Prophylaxis courses	66.5%	55.5%	0.02*
Non-proven infection courses	27.3%	31.3%	0.39
Proven infection courses	6.2%	13.2%	0.02*
Secondary antibiotic courses total	8.2%	6%	0.59
Secondary courses for prophylaxis	2.1%	1.1%	0.68
Secondary courses for non-proven infection	4.1%	4.4%	0.90
Secondary courses for proven infection	2.1%	0.5%	0.37
Infection relapse rate (non-proven + proven)	6.2%	5.0%	0.62

Baseline demographics. *statistically significant, ^a^mean (min/median/max), ^b^median (min-max), ^c^general paediatric surgery, ENT, thoracic surgery.

Regarding possible adverse outcomes due to early discontinuation of antibiotic therapy there was no statistically significant difference in overall mortality, infection-related mortality and infection relapse rate (Tab 1). There was no case of death on our unit during the phase I of the study, whereas 3 children died during phase II, none of them infection related: i) cardiac failure in a newborn baby with critical aortic stenosis, ii) cardiac failure in a baby with unbalanced atrioventricular septum defect and complete AV-block III (palliative care), iii) ischemic necrotizing enterocolitis after cardiopulmonary bypass surgery (antibiotic therapy continued after surgery). Secondary courses of antibiotic therapy during the same hospitalisation after a first course of suspected or proven infection (possible relapses of infection) were even less frequent in phase II. The significantly higher rate of proven infections in phase II was due to an increased rate of pulmonary infections in the winter months November – February compared to phase I in April – June.

## Discussion

As with Patel et al. who evaluated antibiotic use according to the CDC 12-step program on a NICU, we used the CDC recommendations as the evaluation tool for our audit [8,10]. Prolonged empiric antimicrobial treatment without clear evidence of infection, failure to narrow antimicrobial therapy when a causative organism is identified, and no clear documentation about indication and plan for therapy are reported to be common problems, and published data on general inappropriate use of antibiotics have similar findings to our results during phase I [12-16].

The first step in building an intervention for antibiotic surveillance/stewardship is to select the most appropriate strategy that leads to a sustained improvement in a defined area. In the guidelines for developing an institutional program to enhance antimicrobial stewardship there are two core strategies described: 1) prospective audit with feedback and 2) formulary restriction and pre-authorisation [4]. Initiation of antibiotic therapy in the setting of a PICU patient is often empiric and based on apparently worsening clinical condition, with sepsis as a possible factor. During phase I of our audit vancomycin, carbapenems and third generation cephalosporins were not used regularly (not in our top 5 most commonly used antibiotics) and we concluded that the local formulary guidelines appeared to be respected without possible improvement with further formulary restriction and pre-authorisation [16-18]. We therefore selected prospective audit with feedback as a possible intervention, a strategy proposed in a recent review by Newland as the most favourable and efficient [11]. The basis on an audit and feedback intervention is a review of every antibiotic course by a third party (typically an infectious disease physician or a clinical pharmacist) with the audit presented back to the prescriber. For implementation we faced the same barriers reported in other paediatric antibiotic stewardship programs: lack of resources, including funding, time, and personnel [9,11]. We solved this challenge by developing a new strategy for audit and feedback. Instead of personnel audit and feedback by a third party, we created a checklist and used this as a way of audit and feedback (checklist as mandatory form for self-review and feedback by the treating physician). Recent publications about successful use of simple checklists guided us in developing our intervention [19-21]. Key elements for creating a successful checklist are short, clear and basic questions with reminders of routine care [19]. With our checklist, we aimed to support the communication and documentation of indications for the initiation and continuation of antibiotic therapy, which served to remind the treating physician to review and potentially stop therapy or target the isolated pathogen. There are other publications regarding the use of mandatory prescription forms to start antibiotic therapy [22]. The innovation of our approach is that the clinician is reviewing the prescription with help of a checklist at 2 points after start of antibiotic therapy (at 48 hours and 5 days).

The importance of a high compliance rate (power calculation aimed for a compliance rate of 80%) for a successful intervention is emphasised with the statistically not significant increase of antibiotic courses of empiric therapy < 3 days. In contrast to the aviation industry, there is still scepticism by some physicians about the use of checklists for the safe routine care and emergency situations in medicine, illustrated with a citation from A. Gawande in his recently published book “The Checklist Manifesto”: “It runs counter to deeply held beliefs about how the truly great among us – those we aspire to be – handle situations of high stakes and complexity” [19]. A mandatory checklist in a computerised prescription system offers a possible way to improve compliance and impact of the intervention. Due to the potential risk of premature discontinuation of therapy a computerised checklist has to be mandatory but without an automatic antibiotic stop-order [23].

The limitations of our audit were the short surveillance time, the low compliance to the intervention (<80%) and the low numbers of proven infection with positive cultures (no conclusions regarding susceptibility patterns possible). The main strength of our audit was that it was a simple and achievable intervention which was low in cost (no additional funding) and resources. The construction of an easy attainable strategy is very important due to the fact that lack of resources and time are major barriers to implementing an antimicrobial stewardship program in paediatrics.

## Conclusions

The 12 steps of the CDC campaign are a possible tool to evaluate institutional antibiotic therapy. A mandatory checklist as an audit-feedback-mechanism can be a simple achievable intervention with possible improvement in antibiotic use at minimal expense. Further studies with mandatory, computerised checklists within the prescribing system need to be carried out to show compliance and sustainable results over a longer period of time.

## Competing interests

All authors declared that they have not received support (financial and non-financial) from any companies for the submitted work; no authors have any relationships with companies that may have an interest in the submitted work; their spouses, partners, or children.

## Authors’ contributions

MS was responsible for the design of the study, data acquisition and analysis and the final manuscript; FE participated in the study and was responsible for data acquisition; BW was responsible for statistical data analysis and interpretation; JC participated in the study and made substantial contributions to the manuscript; DM helped in the design of the study and made substantial contributions to the manuscript; AF helped in the design of the study, participated in data acquisition and analysis and made substantial contributions to the manuscript. All authors read and approved the final manuscript.

## Pre-publication history

The pre-publication history for this paper can be accessed here:

http://www.biomedcentral.com/1471-2431/12/196/prepub
